# Editorial: Early Intervention in Psychotic Disorders

**DOI:** 10.3389/fpsyt.2020.574532

**Published:** 2020-09-18

**Authors:** Sung-Wan Kim, Barnaby Nelson, Yen Kuang Yang, Young-Chul Chung

**Affiliations:** ^1^ Department of Psychiatry, Chonnam National University Medical School, Gwangju, South Korea; ^2^ Mindlink, Gwangju Bukgu Community Mental Health Center, Gwangju, South Korea; ^3^ Orygen, Parkville, VIC, Australia; ^4^ Centre for Youth Mental Health, The University of Melbourne, Parkville, VIC, Australia; ^5^ Department of Psychiatry, National Cheng Kung University Hospital, College of Medicine, National Cheng Kung University, Tainan, Taiwan; ^6^ College of Medicine, Institute of Behavioral Medicine, National Cheng Kung University, Tainan, Taiwan; ^7^ Department of Psychiatry, Tainan Hospital, Ministry of Health and Welfare, Tainan, Taiwan; ^8^ Department of Psychiatry, Chonbuk National University Medical School, Jeonju, South Korea

**Keywords:** Schizophrenia, psychosis, Ultra-high risk (UHR), clinical high risk (CHR), early intervenction

Psychotic disorders such as schizophrenia are often chronic and disabling in a number of domains, including social and occupational functioning. They typically begin in adolescence or early adulthood, and major changes in the psychosocial functioning of patients with schizophrenia spectrum disorders are often evident within the first 3 years of onset, although the decline in functioning tends to plateau thereafter. Intervention during the early stages of these disorders can reduce their ultimate severity. Therefore, the first 3 years of these disorders have been described as a critical period (1), during which the patient’s future disease course and prognosis are determined.

Over the last twenty years, the retrospective examination of the psychosis prodrome has been replaced by a prospective approach—referred to as the ultra-high risk (UHR) or clinical high risk (CHR) paradigm, with the aim of effectively identifying people who may be at risk of developing a psychotic disorder and, possibly, preventing its progression (2). Early intervention in psychotic disorders generally has two objectives: to prevent the onset of psychotic disorders in people with prodromal symptoms and to provide effective treatment to people in the early stages of psychotic disorder, with the goals of maximizing recovery and reducing the severity of illness (3).

Over the last three decades, numerous studies have investigated the early stages of psychotic disorders. These studies have provided an opportunity to identify factors associated with prevention and treatment outcomes and offer results suggesting how early intervention in psychosis might be the best way to reduce the social and medical burden of schizophrenia (4). However, scientifically derived data addressing many domains of early intervention in psychotic disorders are needed. For example, further research should be conducted to: identify markers for predicting psychotic conversion in UHR patients; investigate the effectiveness of psychosocial intervention and early intervention services; and understand the clinical course and pathogenesis of psychotic disorders beginning at a very early stage of the illness.

This Research Topic covers studies on biomarkers for early psychosis. Hsieh et al. identified a potential biomarker using auditory event-related potentials for individuals at UHR and in their first episode of psychosis. They observed significant sensory gating deficits and impaired deviance detection in this population. Importantly, antipsychotic medications did not seem to impact the sensory gating deficits, making it a convenient marker for the early stages of the psychotic process. Takahashi et al. examined possible relations between pituitary volume and sociocognitive impairments in patients at UHR and with schizophrenia. They found that these subjects had a significantly larger pituitary volume compared to healthy controls, and identified its negative association with working memory in patients with schizophrenia. Berger et al. reported a secondary analysis of the international NEURAPRO clinical trial of omega-3 fatty acids in UHR patients. Their findings indicated that the severity of attenuated psychotic symptoms, general psychopathology, depressive symptoms, and manic symptoms were associated with several classes of fatty acids, partially consistent with previous reports. These findings highlight the possible relevance of membrane fatty acid levels as biomarkers of psychosis risk and also suggest their possible transdiagnostic significance.

Epidemiological studies to investigate factors associated with UHR of psychosis have facilitated our understanding of the pathogenesis and course of UHR of psychosis. Lee et al. reported that problematic Internet use and negative life experiences were significantly associated with psychotic-like experiences in adolescents. Kim et al. evaluated factors associated with psychosocial function and prognostic factors in patients at UHR and with recent-onset schizophrenia. Factor analysis revealed an intrinsic four-factor structure of social-cognitive bias, reflective self, neurocognition, and pre-reflective self. These four factors were found to be associated with baseline social functioning and prodrome-to-psychosis conversion. Conus et al. conducted a questionnaire survey to explore patterns of urban experience, perception when exposed to stressors, and sensitivity to stimuli in early psychosis patients. Findings indicated that city avoidance and negative perceptions toward an urban environment increased in patients after onset of psychosis, suggesting a lower capacity to benefit from the positive aspects of urban spaces. As city avoidance is thought to influence social relations and the recovery process of early psychosis patients, these findings suggest the development of strategies to help patients in their recovery process. Chan et al. conducted a longitudinal study with a follow-up period of 2 years in adolescents and young adults at UHR of psychosis. Low education level, baseline unemployment, a history of violence, and brief limited intermittent psychotic symptoms predicted transition to psychosis, while male sex predicted persistence of the UHR state, or the development of non-psychotic disorders. The results indicated that use of the current UHR criteria can identify individuals who are at imminent risk of developing not just psychosis, but also those who may develop other mental health disorders. Tan et al. examined the longitudinal trend of subjective quality of life among patients with first-episode psychosis to identify the potential influence of patients’ sociodemographic and lifestyle factors. The results indicated that employment was associated with better social relationships and environment, while higher level of educational achievement was associated with improvement of physical health, social relationships, and environment. The results highlighted the need to address educational achievement and employment in the optimization of future early psychosis intervention programs.

Clinical services for the early detection of individuals at clinical high risk of psychosis have been successful in providing psychological interventions and psychosocial support, but the path to vocational recovery has received less attention. Tognin et al. evaluated the presence and quality of educational and employment-focused interventions in the Outreach And Support In South London (OASIS) service, and discussed the path to vocational recovery in these young individuals. They suggested that the focus of early interventions should go beyond alleviating symptoms and move on to the recovery of social and vocational function.

Three review articles discussed cognitive and neurobiological mechanisms in patients at UHR and those with schizophrenia. Yamada et al. performed a systematic review of social cognitive impairment in early psychosis to explore the benefits of early intervention for disturbances of social cognition in psychosis. Allen et al. summarized the significant neurobiological findings in CHR individuals with emergent psychotic symptoms and conversion to psychosis. Delusions were suggested to be related to dysfunction in the medial temporal lobe, particularly the hippocampal–striatal–midbrain network. In addition, disorganized speech and language impairments seemed to be related to lateral temporal dysfunction. Lin et al. reviewed the literature regarding glutamate signal dysfunction, oxidative stress dysregulation, and the links between both in prodromal schizophrenia. Their findings highlighted potential biomarkers related to the *N*-methyl-d-aspartate receptor and oxidative stress regulation.

This Research Topic provides a forum for translational and clinical research conducted in the early stages of psychotic disorders, from the high-risk period to the critical period after the onset of psychosis. Reports published to date have addressed current biological, psychological, and social issues in this field. In addition, they cover the pathogenesis, clinical course, and outcomes of early psychosis. Overall, scientific studies addressing this topic can facilitate our understanding of early psychosis and contribute to the development of effective early intervention strategies.

## Author Contributions

All authors contributed to the article and approved the submitted version.

## Conflict of Interest

The authors declare that the research was conducted in the absence of any commercial or financial relationships that could be construed as a potential conflict of interest.

## References

[1] Birchwood M Todd P Jackson C Early intervention in psychosis. The critical period hypothesis. Br J Psychiatry Suppl (1998) 172:53–9. 9764127

[2] Yung AR McGorry PD McFariane CA Jackson HJ Patton GC Rakkar A Monitoring and care of young people at incipient risk of psychosis. Schizophr Bull (1996) 22:283–303. 10.1093/schbul/22.2.283 8782287

[3] Marshall M Rathbone J Early Intervention for Psychosis. Schizophr Bull (2011) 37:1111–4. 10.1093/schbul/sbr110 PMC3196939 21908851

[4] Fusar-Poli P McGorry PD Kane JM Improving outcomes of first-episode psychosis: an overview. World Psychiatry (2017) 16:251–65. 10.1002/wps.20446 PMC5608829 28941089

